# Anti-inflammatory IL-10 is upregulated in both hemispheres after experimental ischemic stroke: Hypertension blunts the response

**DOI:** 10.1186/2040-7378-5-12

**Published:** 2013-11-13

**Authors:** Abdelrahman Y Fouda, Anna Kozak, Ahmed Alhusban, Jeffrey A Switzer, Susan C Fagan

**Affiliations:** 1 Charlie Norwood VA Medical Center , College of Pharmacy, University of Georgia and Center for Pharmacy and Experimental Therapeutics, Augusta, GA, USA; 2 Department of Neurology, Medical College of Georgia, Georgia Regents University, Augusta, GA, USA

## Abstract

**Background:**

Exogenous administration of the anti-inflammatory cytokine, interleukin 10 (IL-10), is known to promote neuroprotection and mitigate neuroinflammation after ischemia. However, endogenous expression and localization of IL-10 and its receptor (IL-10R) in the post-ischemic brain are still to be elucidated. In this investigation we aimed at determining the temporospatial expression of IL-10 in the rat brain relative to its systemic levels after ischemic stroke.

**Methods:**

Wistar rats were subjected to either permanent (pMCAO) or 3-h temporary (tMCAO) middle cerebral artery occlusion and euthanized at either 24 or 72 h. IL-10/IL-10R levels were quantified in ischemic and contralesional hemispheres and compared to shams using multiplex bead array and Western blotting, respectively. Localization of IL-10/IL-10R with markers for neurons, microglia, astrocytes & endothelial cells were examined using double labeling immunofluorescence. IL-10 was also quantified in the brain tissue of spontaneously hypertensive rats (SHRs) at 24 h after tMCAO.

**Results:**

After both pMCAO and tMCAO in Wistars, IL-10 was significantly upregulated in both hemispheres by ≈ 50% at 24 h while IL-10R expression was significantly decreased only at 72 h in the ischemic hemisphere. IL-10 and IL-10R expression highly co-localized with viable neurons in the ischemic penumbra and contralesional hemisphere. In hypertensive rats, IL-10 showed no significant contralesional upregulation and declined significantly in the ischemic side at 24 h post-ischemia.

**Conclusion:**

Our data highlights the involvement of the ischemic and contralesional neurons in the endogenous anti-inflammatory response after ischemic stroke through increased production of IL-10. This increase in IL-10 is blunted in hypertensive animals and may contribute to worse outcomes.

## Introduction

Innate inflammatory response is a major component of the pathophysiology of ischemic stroke [1,2]. While preclinical studies have shown beneficial effects with adopting an anti-inflammatory approach in the treatment of ischemic stroke, results from clinical trials have been disappointing with no benefit or even worsened outcome in ischemic stroke patients after anti-inflammatory intervention [3,4]. This can be partly explained by the lack of full understanding of the endogenous anti-inflammatory response in the brain following ischemia.

Animal studies have confirmed the anticipated neuroprotective role of the anti-inflammatory cytokine, interleukin 10 (IL-10), in ischemic stroke. Administration of exogenous IL-10 centrally and systemically decreases the infarct size in rats after permanent focal ischemia [5], while IL-10 knockout mice showed larger infarct volume following middle cerebral artery occlusion [6]. Moreover, post-ischemic IL-10 gene transfer attenuated brain infarction in rats subjected to focal and global ischemia [7]. In *in-vitro* models, IL-10 protects murine cortical and cerebellar neurons from excitotoxic damage and oxygen glucose deprivation by activating survival pathways [6,8]. Clinically, lower IL-10 plasma levels have been associated with increased risk of stroke [9]. However, IL-10 is increased in the serum and CSF of patients after ischemic stroke with conflicting reports on its correlation with improved versus worsened outcome [10-12]. It is likely that the ratio of inflammatory versus anti-inflammatory cytokines may give a better picture of overall status in the acute stroke period.

Despite existing knowledge, the temporospatial expression and cellular sources of endogenous IL-10 and its receptor following ischemic stroke are still not known. In the current study, we aimed to elucidate the expression and cellular sources of IL-10 and its receptor in brain tissue of Wistar rats at 24 and 72 h post ischemia. In addition, we examined the change in IL-10 levels after stroke in hypertensive rats.

## Methods

### Middle cerebral artery occlusion (MCAO)

All experimental procedures were approved by the institutional Animal Care and Use Committee of the Charlie Norwood Veterans Affairs Medical Center. 36 Adult male Wistars and 16 spontaneously hypertensive (SHR) rats (270–320 g) underwent permanent (pMCAO) (n = 9), 3 h-temporary (tMCAO) (n = 25) middle cerebral artery occlusion using the suture model, or sham operation (n = 18) as described [13]. Animals were sacrificed at 24 or 72 h and brain tissues were collected for molecular analysis and immunofluorescence.

### Multiplex array system

Cytokine levels in brain homogenates and sera from Wistar rats were simultaneously analyzed using a multiplex array system (Bio-Plex 200; Bio-Rad) according to the manufacturer instructions. After calibrating the instrument, 25 μg for brain and 50 μL of sera were run in triplicates using 4-plex assay kits for tissue and sera respectively and compared to serial dilutions of the standards provided with the kits. Concentrations were determined using the Bio-Plex Manager software program (Bio-Rad version 4.1.1). Cytokine levels below detection limit were assigned a zero value.

### Enzyme linked immunosorbent assay (ELISA)

Brain homogenates from spontaneously hypertensive rats (SHRs) were analyzed for IL-10 using Rat IL-10 sandwich ELISA (RayBiotech) according to the manufacturer’s instructions.

### Western blotting

Brain homogenate aliquots containing 50 μg protein were separated on SDS-PAGE, transferred to nitrocellulose membranes and probed with anti-IL-10Rα rabbit anti-rat antibody (1:500, Santa Cruz) [13]. Protein bands were quantified and normalized to β-actin using ImageJ software.

### Double-labelling immunofluorescence

4 μm paraffin embedded brain sections were processed simultaneously. After rehydration, the sections were boiled in sodium citrate buffer (pH 6.0) for antigen retrieval, permeabilized and blocked in 10% horse serum with 1% BSA in TBS for 2 h at 25°C. For double labeling, two primary antibodies were incubated simultaneously overnight at 4°C at the following dilutions: rabbit anti-IL-10 (1/100; invitrogen), rabbit anti-IL-10Rα (1/100; Santa Cruz), mouse anti-neuronal nuclei (NeuN) (1/100; Millipore) a marker for neurons, mouse anti-glial fibrillary acidic protein (GFAP) (1/300; Sigma-Aldrich) a marker for astrocytes, mouse anti-CD68 (1/100; AbD Serotec) a marker for reactive microglia/macrophages, and goat anti-Von Willebrand factor (VWF) (1/100; Santa Cruz) a marker for endothelial cells. After washing, slides were incubated with fluorescent secondary antibodies, cover slipped with Vectashield mounting medium (Vector Laboratories) and viewed using Zeis Axio Observer.Z1 fluorescent microscope. Negative controls were prepared by omitting the primary antibodies.

### Statistical analysis

The results are expressed as the means ± SEM, and statistical analyses were performed with Student’s t-test using NCSS 2007 software. *P* values less than 0.05 were considered significant.

## Results

### IL-10 is upregulated in both hemispheres at 24 h after MCAO in Wistars

**Figure 1:**
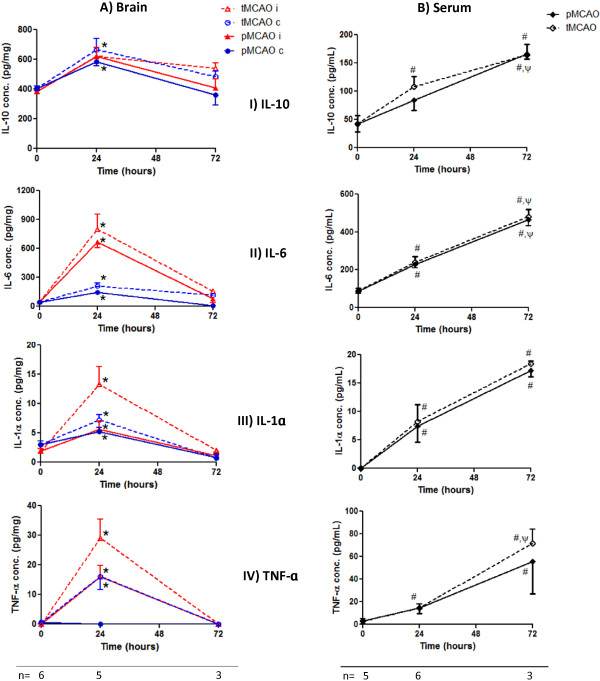
**Cytokines temporal profile in Wistar rat brains and sera after MCAO. (A)** Brain cytokine analysis using Bio-plex 200 showed significant IL-10 upregulation by about 50% in both hemispheres at 24 h compared to baseline. Inflammatory cytokines were strongly increased in the ischemic hemisphere at 24 h then subsided at 72 h. **(B)** Serum cytokine levels showed an ongoing increase at 24 and 72 h. Values are presented as mean ± SEM. i = ischemic hemisphere (red), c = contralesional hemisphere (blue). Solid line represents pMCAO, dashed line represents tMCAO. *, # Significantly different from the corresponding baseline values (p < 0.05), ψ significantly different from the corresponding 24 h values (p < 0.05). Baseline values correspond to sham manipulated animals.

We used the Multiplex array system (Bio-plex 200) to study the cytokine temporal profile in brains and sera of Wistar rats at subacute (24 h) and delayed (72 h) time points in response to MCAO. IL-10 significantly increased by about 50% in both hemispheres at 24 h after both permanent and temporary MCAO. On the other hand, inflammatory cytokines (IL-6, IL-1α & TNF-α) fold increase was much higher in the lesional hemisphere at 24 h with more upregulation after tMCAO then subsided at 72 h (Figure 1A). All cytokine serum levels were significantly upregulated at 24 h and further increased by 2–3 fold at 72 h (Figure 1B).

### Neurons are the main source of IL-10 after MCAO

**Figure 2:**
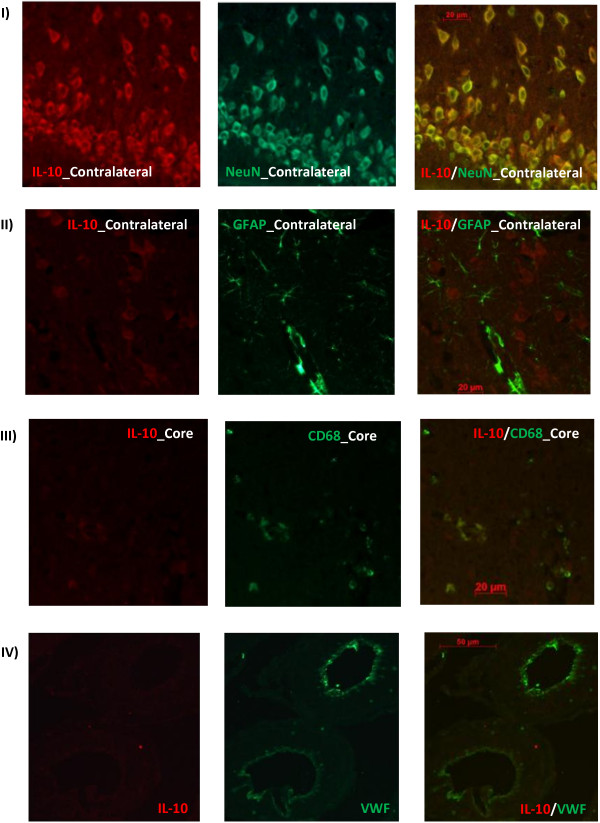
**Immunofluorescent localization of IL-10 in Wistar rat brains after tMCAO.** IL-10 strongly co-localized with NeuN in the ischemic penumbra and contralesional cortical, striatal and hippocampal neurons with less expression in the ischemic core neurons or activated microglia (CD68). IL-10 showed no co-localiztion with markers of astrocytes (GFAP) or endothelial cells (VWF). Images were taken from 24 h brain sections. 72 h brain sections showed similar pattern. N = 4 per group.

Using double labeling immunofluorescence, we explored the spatial expression of IL-10 in sham Wistar rat brains and at 24/72 h after tMCAO using markers for neurons, astrocytes, activated microglia and endothelial cells. IL-10 showed strong co-localization with the neuronal marker, NeuN, in the ischemic penumbra and contralesional cortical, striatal and hippocampal neurons with less expression in the ischemic core neurons or activated microglia (CD68). IL-10 showed no co-localization with markers of astrocytes (GFAP) or endothelial cells (VWF) at the time points studied (Figure 2).

### Temporal and spatial interleukin 10 receptor (IL-10R) protein expression after tMCAO in Wistars

IL-10 executes its pleiotropic actions through binding to IL-10 receptor complex that is composed of two chains, IL-10Rα and IL-10Rβ. Of the two receptor subunits, IL-10Rα is specific for the IL-10 signaling pathway while IL-10Rβ is part of other cytokine receptor complexes as well [14].

**Figure 3:**
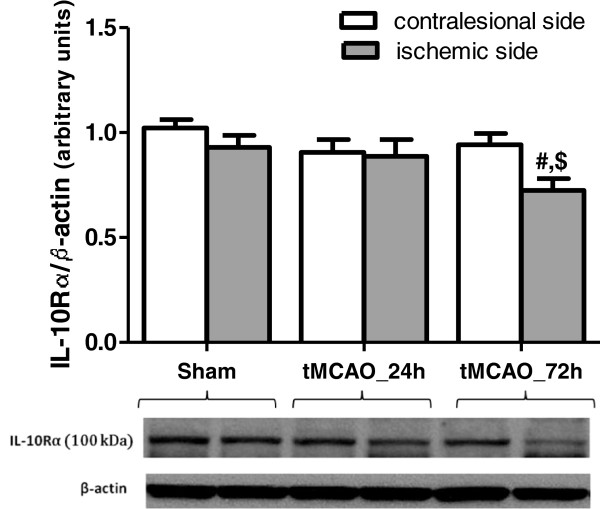
**Temporal IL-10R expression in Wistar rat brains after tMCAO.** Western blotting analysis showed no change in IL-10Rɑ protein expression in Wistars brains at 24 after tMCAO but it declined significantly at 72 h relative to baseline. # significantly different from the sham right hemisphere (p < 0.05), $ significantly different from the 72 h time point contralesional hemisphere (p < 0.05), N = 4-7 per group.

**Figure 4:**
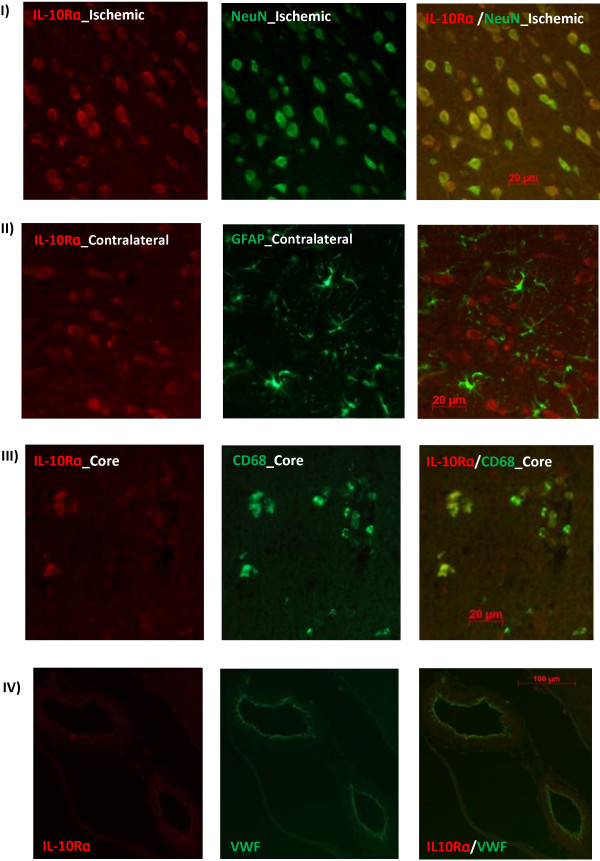
**Immunofluorescent localization of IL-10R in Wistar rat brains after tMCAO.** IL-10Rɑ strongly expressed in neurons (NeuN) and endothelial cells (VWF) of sham and stroked animals in addition to activated microglia/macrophages (CD68) after tMCAO but not astrocytes (GFAP). Images were taken from 24 h brain sections. 72 h brain sections showed similar results. N = 4 per group.

To test whether stroke modulates IL-10Rα expression, we performed Western blotting and immunostaining on Wistar brains subjected to tMCAO. IL-10Rα protein expression did not change at 24 h after tMCAO relative to baseline. However, it significantly declined in the stroked hemisphere at 72 h (Figure 3). Immunflourescent co-localization showed strong IL-10R expression in the neurons (NeuN) and endothelial cells (VWF) of sham operated animals and this expression was extended to activated microglia (CD68) after tMCAO (Figure 4).

### IL-10 upregulation is lost in hypertensive animals after MCAO

**Figure 5:**
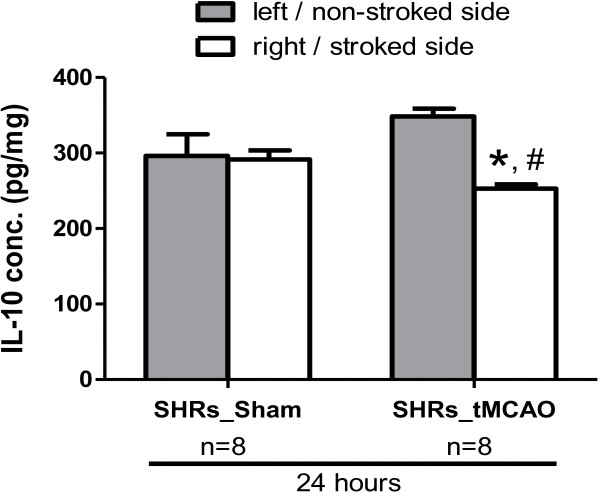
**IL-10 expression in SHR brains at 24 h after tMCAO.** IL-10 levels were quantified in brains of sham and stroked SHR using ELISA. IL-10 was significantly decreased in the ischemic hemisphere of SHRs with a slight non-significant increase contralesionally at 24 h after tMCAO relative to sham values. N = 8 per group. * Significantly different from the sham right hemisphere (p < 0.05), # significantly different from the stroked hemisphere (p < 0.05), N = 8 per group.

Hypertension is associated with a pro-inflammatory milieu. For this reason, we examined the IL-10 levels in SHRs brains after sham operation and at 24 h after 3 h-tMCAO. Baseline IL-10 levels were less in hypertensive rats compared to Wistars. After MCAO, IL-10 was significantly decreased in the stroked hemisphere together with a slight increase in the contralesional hemisphere that was not significant (Figure 5).

## Discussion

This study investigated the endogenous expression of IL-10 and its receptor in the rat brain after experimental stroke. We show, for the first time, that IL-10 is upregulated in both hemispheres at 24 h after both p- and t-MCAO, in contrast to the inflammatory cytokines that were upregulated on the ischemic side. Moreover, immunostaining studies showed neurons to be the major source of IL-10 and its receptor. IL-10 receptor was also expressed in microglia & endothelial cells in accordance with the literature [8,15]. Together with previous reports of localization of IL-6 and its receptor with ischemic brain neurons [16], our data highlights the involvement of neurons in the ischemic inflammatory response being both the source and target of cytokine actions.

The discrepancies in the cytokine levels between brain and serum at 72 h point to the strong involvement of the systemic immune response in cytokine production at this later time point and that serum cytokine levels do not necessarily reflect the local inflammation ongoing in the brain.

Hypertension is a major risk factor for ischemic stroke. The low baseline levels and loss of early upregulation of IL-10 in spontaneously hypertensive rats (SHRs) 24 h after MCAO shows an impaired anti-inflammatory response after ischemic stroke that might be associated with the impaired recovery and worsened outcome in these animals [17].

In summary, our study highlights the contribution of the ischemic and contralesional neurons to the anti-inflammatory response after ischemic stroke through upregulation of IL-10. This outcome is lost under the inflammatory milieu of hypertension. Taken together, these findings suggest that endogenous IL-10 could be a therapeutic target to reduce ischemic damage especially under hypertension. Despite this, the study is limited to the time points examined and does not rule out a possible late role of IL-10 after 3 days post-ischemia involving other brain cells. While a recent study has underscored the functional protective role of IL-10 in ischemic stroke through ameliorating brain inflammation [18], further investigations are needed to examine the mechanisms involved in IL-10 upregulation post-ischemia.

## Competing interests

The authors declare no competing interests.

## Authors’ contributions

AF – Participation in the study design, conduct of experiments, data analysis, and drafting of the manuscript. AK – participation in the study design, conduct of experiments and data analysis. AA – participation in the conduct of experiments and data analysis. JAS – critically revising the manuscript and final edits to the manuscript . SCF – Study design, critically revising the manuscript and final edits to the manuscript. All authors read and approved the final manuscript.
